# Type 2 diabetes mellitus-related changes in left ventricular structure and function in patients with chronic kidney disease

**DOI:** 10.18632/oncotarget.24482

**Published:** 2018-02-13

**Authors:** Pei-Yu Wu, Jiun-Chi Huang, Szu-Chia Chen, Ling-I Chen

**Affiliations:** ^1^ Division of Nephrology, Department of Internal Medicine, Kaohsiung Medical University Hospital, Kaohsiung Medical University, Kaohsiung, Taiwan; ^2^ Department of Internal Medicine, Kaohsiung Municipal Hsiao-Kang Hospital, Kaohsiung Medical University, Kaohsiung, Taiwan; ^3^ Faculty of Medicine, College of Medicine, Kaohsiung Medical University, Kaohsiung, Taiwan

**Keywords:** diabetes mellitus, chronic kidney disease, echocardiographic abnormalities

## Abstract

Type 2 Diabetes mellitus (DM) is the leading cause of chronic kidney disease (CKD) worldwide, and is associated with an increased risk of left ventricular (LV) hypertrophy, LV systolic and diastolic dysfunctions. The aim of this study was to investigate abnormal echocardiographic findings in patients with CKD with and without DM, and identify the factors associated with these abnormalities. We enrolled 356 pre-dialysis patients with CKD (stages 3–5), including 208 with DM and 148 without DM. The structure and systolic and diastolic functions of the left ventricle were assessed using echocardiography, and the clinical and echocardiographic parameters were analyzed. The patients with DM had higher rates of observed/predicted left ventricular mass > 128% (69.5% vs. 56.7%, *p* = 0.015), midwall fractional shortening < 14% (22.6% vs. 8.8%, *p* = 0.001), and ratio of peak early transmitral filling wave velocity to early diastolic velocity of lateral mitral annulus > 12 (32.7% vs. 16.2%, *p* < 0.001) than those without DM. Multivariate analysis showed that male sex, a history of smoking, high systolic blood pressure, high body mass index, high levels of fasting glucose and total cholesterol, low levels of albumin and hemoglobin, and a low estimated glomerular filtration rate were associated with abnormal echocardiographic findings. The rates of inappropriate left ventricular mass, systolic and diastolic dysfunction were higher in our patients with CKD and DM than in those without DM.

## INTRODUCTION

Type 2 diabetes mellitus (DM) is the major cause of chronic kidney disease (CKD) worldwide, and in Taiwanese patients undergoing dialysis it accounts for around 45% of cases of end-stage renal disease. In addition, type 2 DM has been associated with an increased risk of heart failure (HF), morbidity and mortality [1, 2]. However, the reason for this increased risk has yet to be fully elucidated, and cannot be fully explained by traditional risk factors for cardiovascular disease (CVD) [2].

Structural and functional abnormalities of the heart have frequently been reported in patients with renal insufficiency due to pressure and volume overload [3, 4]. In addition, patients with CKD have been associated with high rates of impaired left ventricular (LV) systolic and diastolic function and LV hypertrophy (LVH) [3, 4]. In patients with DM, the most common types of structural and functional heart disease are coronary artery disease (CAD) and diabetic cardiomyopathy, as characterized by LVH, and LV systolic and diastolic dysfunction [5]. Diagnosing preclinical CVD early in this population is important to prevent or delay complications. However, few studies have compared echocardiographic abnormalities in patients with moderate to advanced CKD with and without DM. Therefore, the aims of this study were to compare echocardiographic abnormalities in this population, and evaluate factors associated with these abnormalities in the patients with DM.

## RESULTS

The mean age of the 356 patients was 66.3 ± 12.2 years. Comparisons of the baseline characteristics between the patients with (*n* = 208) and without (*n* = 148) DM are shown in Table 1. The patients with DM had higher rates of hypertension (HTN), CAD, cerebrovascular disease, proteinuria, anti-hypertensive drug treatment including β-blockers, calcium channel blockers and diuretics, higher systolic blood pressure (BP) and higher body mass index (BMI) than those without DM. In addition, the patients with DM had higher levels of fasting glucose and triglycerides, and lower levels of hemoglobin and albumin than those without DM. With regards to the echocardiographic parameters, the patients with DM had higher LV end-systolic volume (LVESV), left atrial (LA) diameter, observed/predicted LV mass (LVM) and peak early transmitral filling wave velocity (E)/early diastolic mitral velocity (Ea), and higher rates of E/Ea > 12, observed/predicted LVM > 128%, midwall fractional shortening (mwFS) < 14%, and LV ejection fraction (LVEF) < 50%, and lower LVEF and mwFS than the patients without DM.

**Table 1:** Comparison of baseline characteristics between patients with and without DM

Characteristics	All patients (*n* = 356)	Without DM (*n* = 148)	With DM (*n* = 208)	*p*
Age (year)	66.3 ± 12.2	66.4 ± 12.9	66.2 ± 11.8	0.914
Male gender (%)	62.9	66.2	60.6	0.278
Smoking history (%)	31.7	32.4	31.3	0.813
Hypertension (%)	83.7	78.4	87.5	0.022
Coronary artery disease (%)	11.8	7.4	14.9	0.031
Cerebrovascular disease (%)	15.2	8.1	20.2	0.002
Systolic blood pressure (mmHg)	142.7 ± 21.8	137.9 ± 20.1	146.0 ± 22.4	0.001
Diastolic blood pressure (mmHg)	79.2 ± 13.1	80.3 ± 12.4	78.3 ± 13.5	0.162
Body mass index (kg/m^2^)	25.5 ± 4.0	24.7 ± 4.1	26.1 ± 3.8	0.002
Laboratory parameters				
Albumin (g/dL)	4.0 ± 0.4	4.1 ± 0.3	4.0 ± 0.4	< 0.001
Fasting glucose (mg/dL)	127.7 ± 60.0	100.3 ± 16.2	147.7 ± 71.3	< 0.001
Triglyceride (mg/dL)	144 (97-200)	123 (89.5-175)	158.5 (105.8-221.5)	< 0.001
Total cholesterol (mg/dL)	196.4 ± 46.4	192.9 ± 43.1	198.9 ± 48.8	0.228
Hemoglobin (g/dL)	11.5 ± 2.3	11.9 ± 2.3	11.3 ± 2.2	0.018
eGFR (mL/min/1.73 m^2^)	25.7 ± 14.2	26.6 ± 15.3	25.0 ± 13.4	0.308
CaXP product (mg^2^/dL^2^)	38.6 ± 9.1	37.8 ± 8.9	39.1 ± 9.3	0.196
Uric acid (mg/dL)	8.1 ± 2.2	7.9 ± 2.1	8.3 ± 2.4	0.095
Proteinuria (%)	66.2	58.5	71.6	0.010
Echocardiographic data				
LVEDV (ml)	118.2 ± 40.2	114.6 ± 31.8	120.7 ± 45.2	0.133
LVESV (ml)	39.5 ± 27.0	35.3 ± 16.5	42.5 ± 32.2	0.006
LAD (cm)	3.8 ± 0.6	3.6 ± 0.6	3.9 ± 0.6	< 0.001
LVMI (g/m^2^)	137.1 ± 47.8	131.4 ± 45.4	141.1 ± 49.1	0.060
LVH (%)	55.1	49.3	59.1	0.067
Observed/predicted LVM (%)	148.3 ± 47.7	140.7 ± 41.1	153.6 ± 51.2	0.011
Observed/predicted LVM > 128% (%)	64.2	56.7	69.5	0.015
LVEF (%)	68.1 ± 10.8	70.0 ± 8.3	66.7 ± 12.1	0.003
LVEF < 50% (%)	5.1	2.0	7.2	0.029
mwFS (%)	16.9 ± 3.6	17.6 ± 2.8	16.3 ± 4.0	< 0.001
mwFS < 14% (%)	16.9	8.8	22.6	0.001
EDT (ms)	224.3 ± 66.1	228.1 ± 67.4	221.6 ± 65.2	0.357
E/Ea	10.3 ± 4.9	9.0 ± 4.3	11.3 ± 5.0	< 0.001
E/Ea > 12 (%)	25.8	16.2	32.7	< 0.001
Medications				
ACEI and/or ARB use	70.2	67.1	72.5	0.280
β-blocker use	32.9	22.6	40.5	< 0.001
Calcium channel blocker use	57.5	47.3	65.0	0.001
Diuretics use	46.0	32.9	55.5	< 0.001

Abbreviations: eGFR, estimated glomerular filtration rate; CaXP product, Calcium-phosphorous product; LVEDV, left ventricular end-diastolic volume; LVESV, left ventricular end-systolic volume; LAD, left atrial diameter; LVMI, left ventricular mass index; LVH, left ventricular hypertrophy; LVM, left ventricular mass; LVEF, left ventricular ejection fraction; mwFS, midwall fraction shortening; EDT, E-wave deceleration time; E, peak early transmitral filling wave velocity; Ea, early diastolic velocity of lateral mitral annulus; ACEI, angiotensin converting enzyme inhibitor; ARB, angiotensin II receptor blocker.

### Determinants of inappropriate LVM and increased observed/predicted LVM

The possible risk factors for an observed/predicted LVM > 128% in the patients with DM in multivariate stepwise analysis are shown in Table 2. The results showed that high BMI (per 1 kg/m^2^, odds ratio [OR], 1.183; 95% confidence interval [CI], 1.071 to 1.307; *p* = 0.001) and low estimated glomerular filtration rate (eGFR) (per 1 mL/min/1.73 m^2^, OR, 0.961; 95% CI, 0.936 to 0.987; *p* = 0.003) were significantly associated with observed/predicted LVM > 128% after adjusting for age, sex, a history of smoking, HTN, CAD, cerebrovascular disease, systolic and diastolic BP, BMI, albumin, fasting glucose, triglycerides, total cholesterol, hemoglobin, eGFR, calcium-phosphorous (CaXP) product, uric acid, proteinuria, and the use of medications including ACEIs, ARBs, β-blockers, calcium channel blockers and diuretics.

**Table 2:** Determinants of abnormal echocardiographic findings of DM patients using binary logistic regression analysis

Echocardiographic findings	Multivariate (stepwise)	
	OR (95% CI)	*p*
Observed/predicted LVM > 128%		
Body mass index (per 1 kg/m^2^)	1.183 (1.071–1.307)	0.001
eGFR (per 1 mL/min/1.73 m^2^)	0.961 (0.936–0.987)	0.003
mwFS < 14%		
Smoking (ever versus never)	2.238 (1.083–4.629)	0.030
Albumin (per 1 g/dL)	0.399 (0.185–0.860)	0.019
E/Ea > 12		
Systolic blood pressure (per 1 mmHg)	1.017 (1.001–1.033)	0.037
Fasting glucose (per 1 mg/dL)	1.006 (1.002–1.011)	0.009
Hemoglobin (per 1 g/dL)	0.640 (0.529–0.774)	< 0.001

Values expressed as odds ratio (OR) and 95% confidence interval (CI). Abbreviations are the same as in Table 1. Covariates in the multivariate model included age, sex, a history of smoking, hypertension, coronary artery disease and cerebrovascular disease, systolic and diastolic blood pressure, body mass index, albumin, fasting glucose, triglyceride, total cholesterol, hemoglobin, eGFR, CaXP product, uric acid, proteinuria, and medications use including ACEI and/or ARB, β-blocker, calcium channel blocker and diuretics.

Multiple stepwise analysis showed that observed/predicted LVM was independently correlated with smoking (ever *vs.* never, unstandardized coefficient β, 16.009; 95% CI, 0.761 to 31.257; *p* = 0.040), BMI (per 1 kg/m^2^, unstandardized coefficient β, 2.089; 95% CI, 0.202 to 3.977; *p* = 0.030), albumin level (per 1 g/dL, unstandardized coefficient β, −19.605; 95% CI, −36.773 to −2.438; *p* = 0.025) and eGFR (per 1 mL/min/1.73 m^2^, unstandardized coefficient β, −0.586; 95% CI, −1.168 to −0.004; *p* = 0.049) (Table 3).

**Table 3:** Determinants of echocardiographic data of DM patients using linear regression analysis

Echocardiographic data	Multivariate (stepwise)	
	Unstandardized coefficient β (95% CI)	*p*
Observed/predicted LVM		
Smoking (ever versus never)	16.009 (0.761 31.257)	0.040
Body mass index (per 1 kg/m^2^)	2.089 (0.202, 3.977)	0.030
Albumin (per 1 g/dL)	−19.605 (−36.773, −2.438)	0.025
eGFR (per 1 mL/min/1.73 m^2^)	−0.586 (−1.168, −0.004)	0.049
mwFS		
Male versus female	−1.493 (−2.641, −0.345)	0.011
Albumin (per 1 g/dL)	1.956 (0.679, 3.234)	0.003
Total cholesterol (per 1 mg/dL)	−0.014 (−0.025, −0.002)	0.019
E/Ea		
Systolic blood pressure (per 1 mmHg)	0.036 (0.008, 0.063)	0.012
Albumin (per 1 g/dL)	−1.852 (−3.331, −0.373)	0.014
Fasting glucose (per 1 mg/dL)	0.009 (0.000, 0.017)	0.046
Hemoglobin (per 1 g/dL)	−0.728 (−1.042, −0.413)	< 0.001
β-blocker use	1.528 (0.247, 2.810)	0.020
Diuretics use	1.417 (0.102, 2.732)	0.035

Values expressed as unstandardized coefficient β and 95% confidence interval (CI). Abbreviations are the same as in Table 1. Covariates in the multivariate model included age, sex, a history of smoking, hypertension, coronary artery disease and cerebrovascular disease, systolic and diastolic blood pressure, body mass index, albumin, fasting glucose, triglyceride, total cholesterol, hemoglobin, eGFR, CaXP product, uric acid, proteinuria, and medications use including ACEI and/or ARB, β-blocker, calcium channel blocker and diuretics.

### Determinants of mwFS < 14% and decreased mwFS

Multivariate stepwise analysis (Table 2) also revealed that smoking (ever *vs.* never, OR, 2.238; 95% CI, 1.083 to 4.629; *p* = 0.030) and a low albumin level (per 1 g/dL, OR, 0.399; 95% CI, 0.185 to 0.860; *p* = 0.019) were significantly associated with mwFS < 14%.

Multiple stepwise analysis showed that male sex (*versus* female sex, unstandardized coefficient β, −1.493; 95% CI, 2.641 to −0.345; *p* = 0.011), albumin level (per 1 g/dL, unstandardized coefficient β, 1.956; 95% CI, 0.679 to 3.234; *p* = 0.003), and level of total cholesterol (per 1 mg/dL, unstandardized coefficient β, −0.014; 95% CI, −0.025 to −0.002; *p* = 0.019) were significantly correlated with mwFS (Table 3).

### Determinants of E/Ea > 12 and increased E/Ea

Multivariate stepwise analysis showed significant correlations between high systolic BP (per 1 mmHg, OR, 1.017; 95% CI, 1.001 to 1.033; *p* = 0.037), high fasting glucose level (per 1 mg/dL, OR, 1.006; 95% CI, 1.002 to 1.011; *p* = 0.009) and low hemoglobin level (per 1 g/dL, OR, 0.640; 95% CI, 0.529 to 0.774; *p* < 0.001) and E/Ea > 12 in the patients with DM (Table 2).

In addition, multiple stepwise analysis showed that E/Ea was independently correlated with systolic BP (per 1 mmHg, unstandardized coefficient β, 0.036; 95% CI, 0.008 to 0.063; *p* = 0.012), levels of albumin (per 1 g/dL, unstandardized coefficient β, −1.852; 95% CI, −3.331 to −0.373; *p* = 0.030), fasting glucose (per 1 mg/dL, unstandardized coefficient β, 0.009; 95% CI, 0.000 to 0.017; *p* = 0.046), and hemoglobin (per 1 g/dL, unstandardized coefficient β, −0.728; 95% CI, −1.042 to −0.413; *p* < 0.001), and the use of β-blockers (unstandardized coefficient β, 1.528; 95% CI, 0.247 to 2.810; *p* = 0.020) and diuretics (unstandardized coefficient β, 1.417; 95% CI, 0.102 to 2.732; *p* = 0.035) (Table 3).

## DISCUSSION

In this study, we compared echocardiographic abnormalities among patients with CKD stage 3–5 with and without DM, and evaluated the factors associated with these abnormalities in the patients with DM. Our analysis showed that the patients with DM had higher rates of abnormalities including increased LA diameter, inappropriate LVM, and LV systolic and diastolic dysfunction. Furthermore, male sex, smoking, high systolic BP, high BMI, hyperglycemia, a high level of total cholesterol, low levels of hemoglobin and albumin, and low eGFR were associated with abnormal echocardiographic findings in the patients with CKD and DM.

The worse echocardiographic findings including inappropriate LVM, and LV systolic and diastolic dysfunction in the patients with CKD and DM compared to those without DM is an important finding of this study. Hartog et al. suggested the importance of advanced glycation endproducts (AGEs) with regards the pathobiology of HF, in that they may impair both diastolic and systolic LV function directly through extracellular matrix protein cross-linkage and/or altered calcium handling [6]. Furthermore, AGEs may indirectly impair diastolic and systolic LV function through interactions with cardiac AGE receptors, thereby causing low-grade inflammation, increased oxidative stress and altered gene expressions [6, 7]. Therefore, it is possible that AGEs have a negative impact on LV remodeling [6, 7]. This impact may be especially relevant in patients with type 2 DM due to chronic hyperglycemia and hyperlipidemia which can induce the formation of AGEs and also oxidative stress and low-grade inflammation, thereby leading to increased production of AGEs [6–8].

### LVH and inappropriate LVM

The reported prevalence of LVH in patients with type 2 DM ranges from 19% to 56% in different studies [9–11]. In the present study, the prevalence of LVH was 59.1% in the patients with type 2 DM, which may have been due to the high prevalence of HTN (87.5%) in our study cohort with CKD. In addition, the patients with DM also had a high prevalence of inappropriate LVM (69.5%). Various alterations in hemodynamic and metabolic function have been reported to affect the structure and function of the heart in patients with CKD, and an excessive increase in LVM, a condition termed ‘inappropriate LVM’, has been reported in patients with CKD to compensate for these alterations [12, 13]. Several studies have reported that predicted LVM according to the sex, height^2.7^, and hemodynamic load of a patient can be used to assess inappropriate LVM, and that the appropriateness of LVM can be estimated by the ratio of observed to predicted LVM [14, 15]. Recent studies have reported that inappropriate LVM and LVH occur in a significant proportion of patients with arterial HTN and aortic stenosis, and that this has a negative impact on the prognosis of CVD [16–20].

### LV systolic dysfunction

Most previous studies have reported a high rate of LV systolic dysfunction in patients with DM, ranging from 5% to 21.8% [9, 10, 21]. LV systolic function can be estimated using conventional methods such as LVEF or by methods independent of geometry such as mwFS. Both of these systolic parameters have been reported to be good predictors of cardiovascular events in patients with renal failure [22]. In the present study, the rates of LVEF < 50% and mwFS < 14% were 7.2% and 22.6%, respectively, which is consistent with previous studies.

### LV diastolic dysfunction

LV diastolic dysfunction has been reported to occur in 25% to 75% of patients with diabetes [9, 10, 23–25]. We used the E/Ea ratio to define diastolic dysfunction according to tissue Doppler imaging. It has been reported that not performing tissue Doppler imaging can exclude up to 25% of patients with pseudonormalization which may then go unnoticed [10]. The E/Ea ratio has been reported to be significantly correlated with both LV diastolic function and filling pressure [26, 27]. In addition, an enlarged LA has been reported to be a marker of adverse cardiovascular outcomes including stroke, congestive HF, atrial fibrillation, and cardiovascular death in various pathologic conditions [28, 29]. Both pressure and volume overload can contribute to enlargement of the left artery, and patients with a large left artery may have impaired LV diastolic function [28]. In the present study, the patients with CKD and DM had a larger LA diameter, higher E/Ea ratio, and higher rate of E/Ea > 12 compared to those without DM.

There are several limitations to this study. First, we excluded patients with mitral valve disease, atrial fibrillation, and inadequate image visualization. Hence, our results cannot be applied to these patients, and the generalizability of the results is limited. Second, we included patients with DM regardless of the duration of DM, and a longer duration can result in more diverse effects on heart function. Therefore, we could not assess the effect of the duration DM on echocardiographic findings. Third, this study was cross-sectional, and subsequent clinical prognosis could not be confirmed. Future study of clinical outcomes needed to be addressed. In addition, we did not quantitatively assess proteinuria, so could not fully analyze the impact of proteinuria. Finally, antihypertensive drug treatment can influence the geometry and functional parameters of the left ventricle. However, due to ethical concerns, we did not withhold any drugs at the time of the echocardiography evaluations. However, in order to investigate the influence of drugs, we included various classes of antihypertensive drugs in the analysis. Our results showed that β-blockers and diuretics were associated with an increased E/Ea ratio, however this may have been due to selection bias.

In conclusion, in our study cohort of patients with stage 3–5 CKD, the prevalence of echocardiographic abnormalities including inappropriate LVM, and LV systolic and diastolic dysfunction was higher in the patients with DM. Traditional cardiovascular risk factors including male sex, smoking, HTN, obesity, hyperglycemia, and dyslipidemia, and nontraditional risk factors including malnutrition, anemia and decreased renal function were associated with abnormal echocardiographic findings in the patients with CKD and DM.

## MATERIALS AND METHODS

### Study patients and design

This study was conducted at the Outpatient Department of Internal Medicine at a regional hospital in southern Taiwan, and enrolled 356 patients with CKD stages 3 to 5 from January 2007 to May 2010. Patients with evidence of kidney damage for more than 3 months were classified into three groups according to the stage of CKD as assessed by eGFR as recommended in the National Kidney Foundation-Kidney Disease Outcomes Quality Initiative (K/DOQI) guidelines [30] as follows: stage 3, 30 to 59 mL/min/1.73 m^2^; stage 4, eGFR 15 to 29 mL/min/1.73 m^2^; and stage 5, eGFR < 15 mL/min/1.73 m^2^. We excluded patients with significant mitral valve disease and images of insufficient quality, and also those receiving dialysis. The study protocol was approved by the Institutional Review Board of Kaohsiung Medical University Hospital, and all participants provided written informed consent to participate in this study. The methods were carried out in accordance with the approved guidelines.

### Evaluation of cardiac structure and function

All echocardiographic examinations were performed by two experienced cardiologists using a VIVID 7 system (General Electric Medical Systems, Horten, Norway), with the patient lying quietly in the left decubitus position. The cardiologists were blinded to the patients’ clinical data. Two-dimensional and two-dimensionally guided M-mode images were recorded from standardized views. Echocardiographic parameters including LV end-diastolic volume, LVESV, LA diameter, LV internal diameter in diastole (LVIDd), LV posterior wall thickness in diastole (LVPWTd), interventricular septal wall thickness in diastole (IVSTd), E, Ea, and E/Ea ratio were recorded. LV systolic function was assessed according to LVEF and mwFS [31]. The LVM was calculated using a modification of Devereux's method: LVM = 1.04 × [(IVSTd + LVIDd + LVPWTd)^3^ – LVIDd^3^] – 13.6g [32]. LVMI was calculated as LVM/body surface area, and LVH was defined according to the 2007 European Society of Hypertension/European Society of Cardiology guidelines [33]. Inappropriate LVM was assessed according to the ratio of observed to predicted LVM, where predicted LVM = 55.37 + 6.64 × height (m^2.7^) + 0.64 × stroke work - 18.07 × sex (male = 1, female = 2) [14]. Stroke work was estimated as systolic BP × volume, and converted to gram meters by multiplying by 0.0144. ‘Inappropriate’ LVM was defined as an observed value more than 28% of the predicted value (i.e. observed/predicted LVM > 128%) [14, 15]. Raw ultrasound data were recorded and analyzed offline by a cardiologist blinded to other data using EchoPAC software (GE Medical Systems). Echocardiographic data were recorded from three consecutive beats and then averaged.

### Collection of demographic, medical, and laboratory data

Data including age, sex, smoking history (ever *vs.* never), and comorbidities were recorded from the patients’ medical records or interviews. BMI was calculated as the weight in kilograms divided by the height in meters squared. Blood and urine samples were obtained from the patients within 1 month of enrollment into the study. and the laboratory data were obtained from fasting blood samples using an autoanalyzer (Roche Diagnostics GmbH, D-68298 Mannheim COBAS Integra 400). Levels of serum creatinine were measured using the compensated Jaffé method (kinetic alkaline picrate) using a Roche/Integra 400 Analyzer (Roche Diagnostics, Mannheim, Germany) with a calibrator that could be traced in isotope-dilution mass spectrometry [34]. The eGFR was calculated using the equation reported in the Modification of Diet in Renal Disease (MDRD) study [35]. A positive result was defined as a score of 1+ or more. Data regarding the use of medications including angiotensin converting enzyme inhibitors (ACEIs), angiotensin II receptor blockers (ARBs), β-blockers, calcium channel blockers, and diuretics during the study period were recorded from the patients’ medical records.

### Statistical analysis

All statistical analyses were performed using SPSS software for Windows version 19.0 (SPSS Inc. Chicago, USA). Data were expressed as percentages, means ± standard deviations, and medians (25th-75th percentile) for triglycerides. Between-group differences were assessed using the chi-square test for categorical variables and the independent *t*-test for continuous variables. Multivariate binary logistic analysis and linear regression analysis were used to identify factors associated with abnormal echocardiographic findings in the patients with CKD and DM. Covariates included in the multivariate model included age, sex, smoking history, HTN, CAD, cerebrovascular disease, systolic and diastolic BP, eGFR, proteinuria and BMI, and levels of albumin, fasting glucose, triglycerides, total cholesterol, hemoglobin, CaXP product and uric acid, as well as the use of medications including ACEIs, ARBs, β-blockers, calcium channel blockers and diuretics. A *p* value of less than 0.05 was considered to indicate statistical significance.

## Abbreviations

DM: Diabetes mellitus
CKD: chronic kidney disease
HF: heart failure
CVD: cardiovascular disease
LV: left ventricular
LVH: LV hypertrophy
CAD: coronary artery disease
HTN: hypertension
BP: blood pressure
BMI: body mass index
LVESV: LV end-systolic volume
LA: left atrial
LVM: LV mass
Ea: peak early transmitral filling wave velocity (E) early diastolic mitral velocity
mwFS: midwall fractional shortening
LVEF: LV ejection fraction
eGFR: estimated glomerular filtration rate
CaXP: calcium-phosphorous
AGEs: glycation endproducts
LVIDd: LV internal diameter in diastole
LVPWTd: LV posterior wall thickness in diastole
IVSTd: interventricular septal wall thickness in diastole
ACEIs: angiotensin converting enzyme inhibitors
ARBs: angiotensin II receptor blockers
